# End-to-Side Reconstruction for Two Renal Arteries Affects the Recovery of the Graft Function in Living Donor Renal Transplantation

**DOI:** 10.7759/cureus.93110

**Published:** 2025-09-24

**Authors:** Ryota Masui, Shigeyoshi Yamanaga, Mariko Toyoda, Chiaki Kawabata, Yoei Miyabe, Yasuhiro Yamamoto, Yu Watanabe, Akito Inadome, Yuji Hidaka, Hiroshi Yokomizo

**Affiliations:** 1 Department of Surgery, Japanese Red Cross Kumamoto Hospital, Kumamoto, JPN; 2 Department of Nephrology, Japanese Red Cross Kumamoto Hospital, Kumamoto, JPN; 3 Department of Urology, Japanese Red Cross Kumamoto Hospital, Kumamoto, JPN

**Keywords:** graft function, graft survival, living donor renal transplantation, multiple artery, renal artery

## Abstract

Background/Objectives: Kidneys with multiple arteries are occasionally encountered in living-donor renal transplantation. However, the impact of different reconstruction methods on graft outcomes remains unclear. Therefore, we aimed to analyze the effect of the end-to-side reconstruction technique on short- and long-term outcomes in living donor renal transplantation.

Methods: We retrospectively analyzed 254 living donor renal transplants performed at our center between 2011 and 2024. Sixty patients with dual renal arteries were categorized into five groups based on the type of reconstruction method: end-to-side (n=5, 2.0%), inferior epigastric artery (n=15, 5.9%), direct anastomosis (n=2, 0.79%), side-to-side (n=29, 11.4%), and ligated (n=9, 3.5%). Furthermore, we compared the incidence of early postoperative events and long-term survival with those of a control group of single-artery transplants. Long-term survival was analyzed using Kaplan-Meier and Cox regression analyses. Cox regression was adjusted for several factors.

Results: The end-to-side group had a significantly higher incidence of delayed graft function recovery (p<0.001) and a trend toward increased all-cause graft loss (p=0.254) compared with the control group, whereas other groups showed no significant difference.

Conclusions: Our study showed a higher incidence of delayed graft renal function recovery and inferior long-term graft survival only in the end-to-side group. Further multicenter studies are required to evaluate alternative reconstruction techniques.

## Introduction

Approximately 7-28% of kidney transplant cases involve renal arteries, often requiring a revascularization technique in living-donor renal transplantation [1]. Various reconstruction techniques are typically used to create an orifice on the back table before anastomosis [2]. A meta-analysis reported that, owing to the technical challenges, kidneys with multiple renal arteries have a higher risk of surgical complications and delayed graft function than those with a single artery. However, long-term graft survival and patient survival are reportedly equivalent [1]. Conversely, only a few studies have examined whether a specific type of reconstruction technique affects graft and recipient outcomes [3,4].

Given the scarcity of deceased donations, living-donor kidney transplantation is widely performed in Japan [5], and the opportunity for a second transplantation is extremely limited. Thus, maximizing the success of the primary transplant is crucial. In this study, we aimed to validate the impact of the end-to-side reconstruction technique on short- and long-term outcomes in living-donor renal transplantation.

## Materials and methods

Patient population

We retrospectively analyzed 267 living-donor renal transplantations performed at Japanese Red Cross Kumamoto Hospital, Kumamoto, Japan, between 2011 and 2024. The donor kidneys were retrieved laparoscopically. Furthermore, to assess the genuine impact of each reconstruction method, we excluded 13 cases: kidneys with more than two arteries (n=8), cases with hyperacute rejection (n=2), cases reconstructed using an internal iliac artery graft (n=1), and cases with a follow-up period of less than one month after transplant (n=2). Sixty cases with two renal arteries were divided into five groups based on the reconstruction method and compared with a single-artery group (n=194) as a control. The standard immunosuppressive regimen included tacrolimus, mycophenolate mofetil (MMF), and methylprednisolone, while desensitization therapy consisted of rituximab, plasmapheresis, and MMF, as described previously [6]. This study was approved by the Institutional Review Board of the Japanese Red Cross Kumamoto Hospital (approval number 641). The requirement for informed consent was waived owing to the retrospective nature of the study. 

Details of revascularization techniques

**Figure 1:**
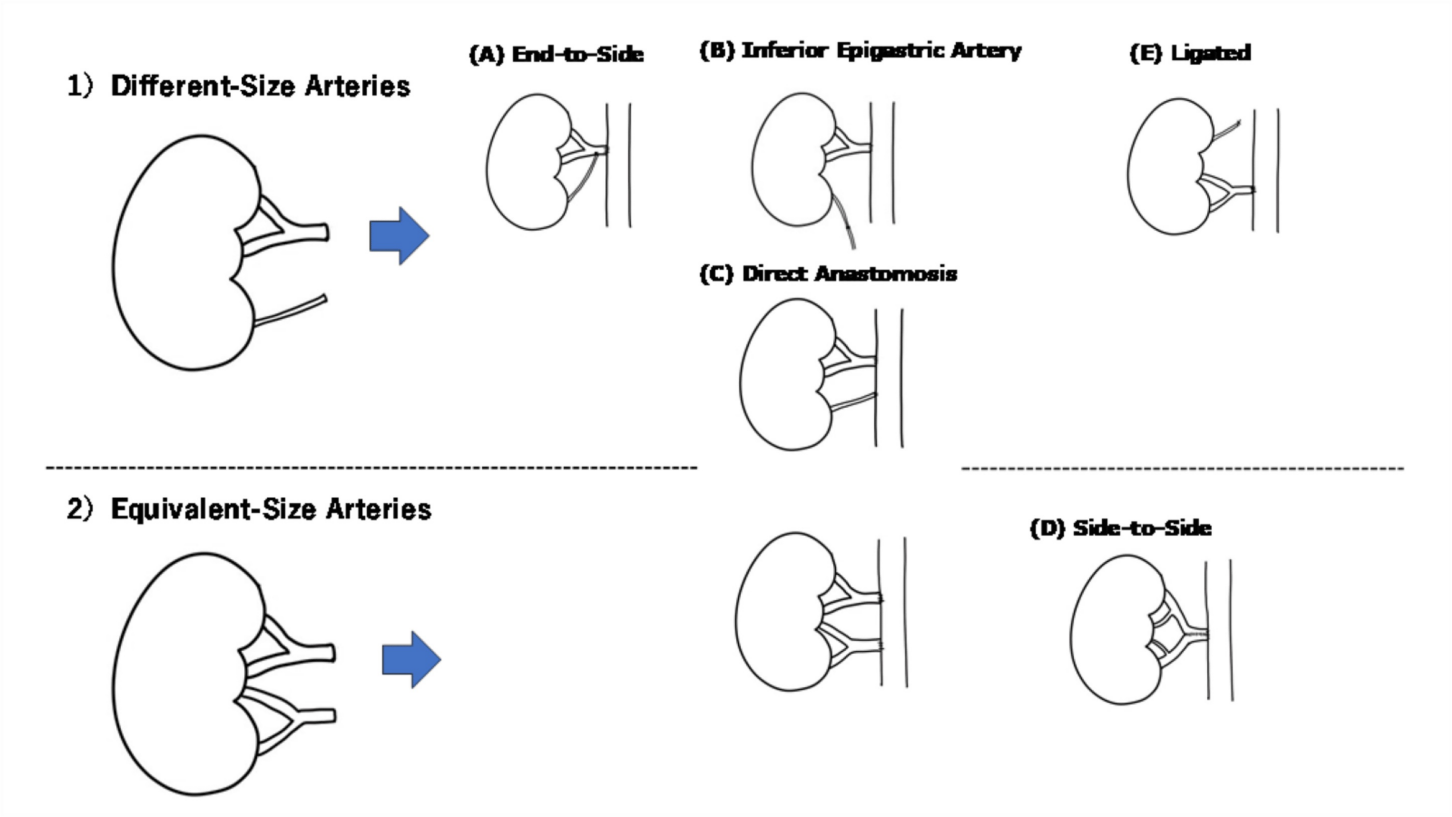
Details of revascularization techniques for the kidneys with two renal arteries. First author Ryota Masui drew this figure.

Figure 1 shows the images of each revascularization technique. In cases where the two renal arteries differed significantly in size, the following techniques were selected: (A) end-to-side suturing the end of the smaller artery to the side of the dominant artery; (B) inferior epigastric artery-anastomosing the smaller artery to the recipient inferior epigastric artery; and (C) direct anastomosis-anastomosing each artery directly to the external/common iliac artery. (E) Ligation is the method of sacrificing the smaller artery and is only applied when the diameter of the smaller artery is < 2 mm and/or feeding < 10% of the whole renal cortex. Notably, lower pole arteries were always revascularized, as they are critical for ureteral blood supply. If the sizes of the two arteries were equivalent, direct anastomosis was performed as described above, and the side-to-side technique (D) (the two arteries were joined into a single orifice and anastomosed to the external/common iliac artery) was selected.

Outcomes

We compared the donor and recipient baseline characteristics, early postoperative events, and overall graft survival of each reconstruction technique with those of the control group. The early postoperative events included delayed graft function (DGF), slow graft function (SGF), ureteral complications, acute rejection, and thrombotic events. DGF was defined as the temporary need for hemodialysis within seven days postoperatively, and SGF was defined as a serum creatinine level > 3 mg/dL on postoperative day 4.

Statistical analyses

The chi-square test was used for categorical data, and the Kruskal-Wallis test was used for continuous data. Post-hoc tests for categorical and continuous data were adjusted using the Bonferroni method. Continuous data was expressed as median (first quartile-third quartile), and statistical significance was set at a two-tailed p-value <0.05. The Kaplan-Meier method was used to analyze overall graft survival. Cox regression analysis was used to analyze the impact of each reconstruction technique on survival, adjusted for age, sex of donor and recipient, kidney side, transplant period, recipient surgeon, operation time, and total ischemic time. All analyses were performed using IBM SPSS Statistics for Windows, Version 28 (Released 2021; IBM Corp., Armonk, New York, United States).

## Results

Baseline characteristics

Recipient ages ranged from 15 to 73 years, with no cases of retransplantation. For some reconstruction techniques, donor and recipient operation times were significantly longer compared to those in the control group (Table 1). The total ischemic time was significantly longer in the side-to-side group. The donor and recipient sexes differed significantly between some reconstruction techniques. However, other characteristics, including age and body mass index, did not differ between groups.

**Table 1:** Baseline characteristics For categorical data, the test value was the chi-square statistic, while for continuous data, the test value was the Kruskal-Wallis test statistic (H). *p<0.05 after post-hoc test by Bonferroni method (vs single artery). BMI, body mass index

Parameters	Single Artery (n=194)	End-to-Side (n=5)	Inferior Epigastric Artery (n=15)	Direct Anastomosis (n=2)	Side-to-Side (n=29)	Ligated (n=9)	Test values	p-value
Donor age, years, median (1st-3rd quartile)	58 (51-64)	59 (48-69)	54.5 (45.5-67)	58.5 (52-65)	60 (53.5-66.5)	55 (48-60)	2.14	0.82
Donor Sex, n(%)							12.03	0.034
Female	134(69.1)	4(80)	15(100)	1(50)	15(51.7)	5(55.6)		
Male	60(30.9)	1(20)	0(0)	1(50)	14(48.3)	4(44.4)		
Donor BMI, kg/m², median (1st-3rd quartile)	23 (20.8-24.9)	23.1 (20.3-26.9)	21.5 (20.75-22.75)	25.6 (24.2-27)	23.1 (21.25-26)	23.8 (22.3-24.2)	4.73	0.45
Side of kidneys, n(%)							5.16	0.39
Left	169(87.1)	5(100)	13(86.7)	2(100)	23(79.3)	6(66.7)		
Right	25(12.9)	0(0)	2(13.3)	0(0)	6(20.7)	3(33.3)		
Donor serum Cr at baseline, mg/dl, median (1st-3rd quartile)	0.66 (0.57-0.73)	0.67 (0.6-0.69)	0.62 (0.53-0.64)	0.6 (0.53-0.66)	0.68 (0.62-0.77)	0.64 (0.56-0.79)	7.85	0.16
Donor operation time, minutes, median (1st-3rd quartile)	192 (176-208)	206 (189-240)	190 (172-207)	236 (209-263)	208 * (197-228)	219 (204-233)	16.34	0.006
Recipient age, years, median (1st-3rd quartile)	49 (36-59)	48 (43-53)	57 (50-65.5)	41 (28-54)	47 (39-58.5)	34 (21-52)	6.39	0.27
Recipient Sex, n(%)							11.93	0.036
Female	66(34)	0(0)	1(6.7)	2(100)	10(34.5)	4(44.4)		
Male	128(66)	5(100)	14(93.3)	0()	19(65.5)	5(55.6)		
Recipient BMI, kg/m², median (1st-3rd quartile)	21.9 (19.6-25.1)	23.3 (20.4-26.7)	22.55 (19.25-25.8)	18.7 (18.4-19)	23.2 (21.7-25.55)	22.3 (20.1-24.1)	4.49	0.48
Duration of dialysis, months, median (1st-3rd quartile)	7 (0-27)	10 (0-45)	1 (0-43)	0 (0)	12 (2-24)	17 (0-73)	4.13	0.53
Recipient operation time, min, median (1st-3rd quartile)	340 (306-374)	443 * (418-475)	385 * (379-430)	409 (371-448)	409 * (384-430)	374 (341-374)	59.43	<0.001
ABO blood type incompatible, n(%)	60(30.9)	3(60)	4(26.7)	1(50)	6(20.7)	5(55.6)	6.40	0.26
Length of follow up, months, median (1st-3rd quartile)	68 (33-110)	86 (83-91)	77.5 (57-116.5)	66.5 (66-67)	102 (60.5-127)	56 (55-118)	8.34	0.13
Transplant period, n(%)							16.31	0.091
2011-2014	51(26.3)	3(60)	4(26.7)	0(0)	13(44.8)	4(44.4)		
2015-2019	75(38.7)	2(40)	8(53.3)	2(100)	12(41.4)	4(44.4)		
2020-2024	68(35.1)	0(0)	3(20)	0(0)	4(13.8)	1(11.1)		
Total ischemic time, minutes, median (1st-3rd quartile)	95 (82-110)	128 (113-131)	113 (91-129)	111 (103-119)	142 * (124-149)	119 (100-127)	56.25	<0.001
Warm ischemic time, seconds, median (1st-3rd quartile)	219 (186-258)	242 (194-303)	215 (184-284)	186 (184-189)	236 (214-291)	223 (202-262)	6.22	0.28

Early postoperative events

The end-to-side group had a significantly higher incidence of DGF or SGF (Table 2). However, the incidence of ureteral complications and acute rejection did not differ significantly between the groups. Thrombotic events did not occur in any of the groups.

**Table 2:** Recipient complications after transplantation The chi-square test was used .*p<0.05 after post-hoc test by Bonferroni method (vs single artery). DGF, delayed graft function; SGF, slow graft function.

Events	Single Artery	End-to-Side	Inferior Epigastric Artery	Direct Anastomosis	Side-to-Side	Ligated	Chi-square Value	p-value
Acute rejection, n(%)	11(5.7)	0(0)	0(0)	0(0)	0(0)	0(0)	3.56	0.61
Ureteral complication, n(%)	4(2.1)	0(0)	0(0)	0(0)	0(0)	0(0)	1.26	0.93
DGF or SGF, n(%)	4(2.1)	2(40) *	1(6.7)	0(0)	2(6.9)	0(0)	22.48	<0.001
Thrombosis, n(%)	0(0)	0(0)	0(0)	0(0)	0(0)	0(0)	-	-

Analyses of long-term survival

**Figure 2:**
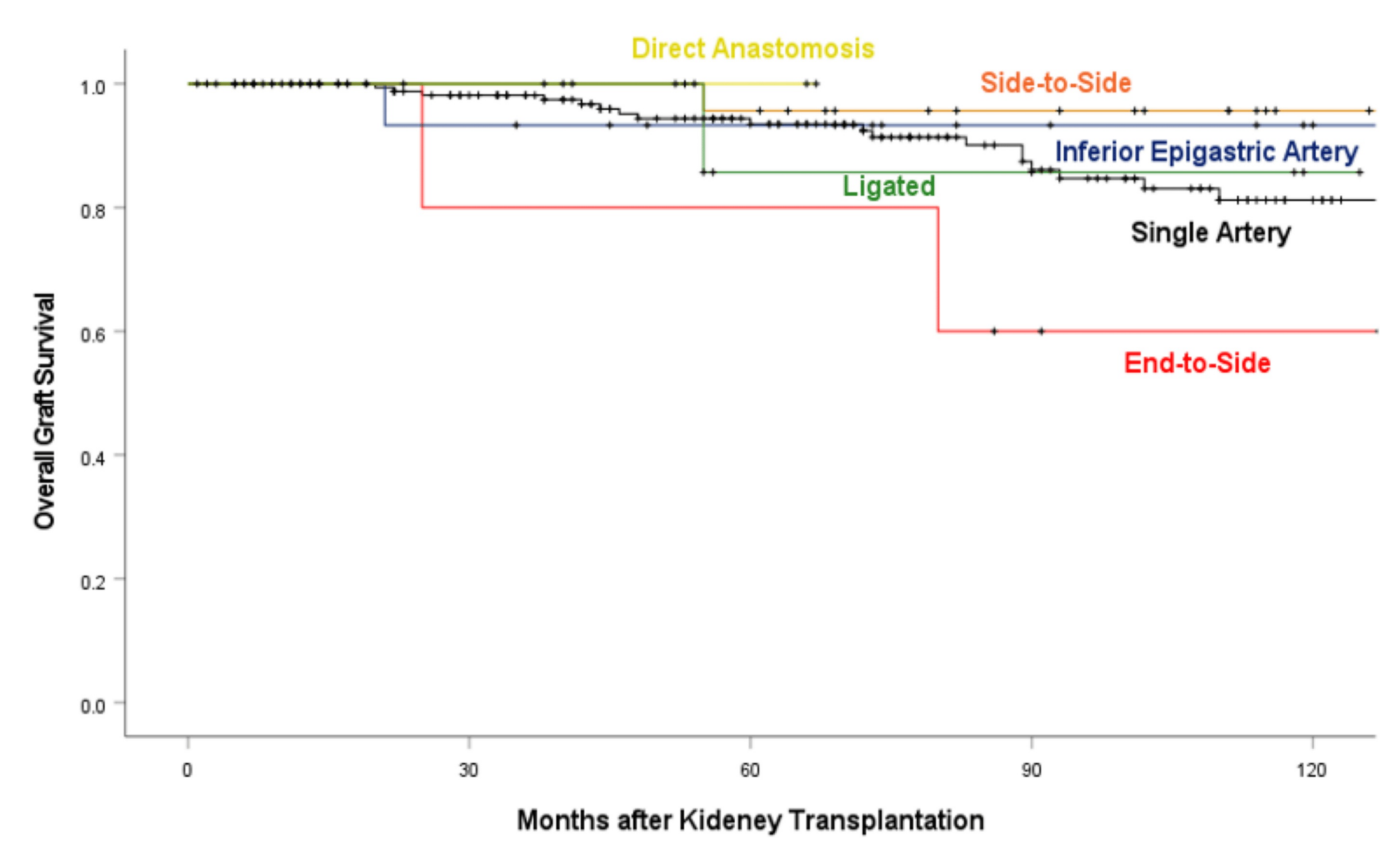
Kaplan-Meier analysis for overall graft survival

The five- and 10-year overall graft survival rates for the end-to-side group were the lowest among all groups, though the difference was not statistically significant (Figure 2 and Tables 3, 4). The outcomes of the other reconstruction techniques were equivalent to those of the single-artery group. After multivariate adjustment for age, sex of donor and recipient, side of the kidney, transplant period, recipient surgeon, operation time, and total ischemic time, the end-to-side group showed a higher adjusted hazard ratio for all-cause graft loss (Table 5).

**Table 3:** Number at risk for the Kaplan-Meier curve

	0 days	30 days	60 days	90 days	120 days
Single Artery	194	149	104	63	32
End-to Side	5	4	4	2	1
Inferior Epigastric	15	14	11	5	1
Direct Anastomosis	2	2	2	1	1
Side-to-Side	29	27	22	16	8
Ligated	9	9	4	3	1

**Table 4:** 5- and 10-year overall graft survival for each group The p-value was 0.291, as determined by the log-rank test.

	Single Artery	End-to Side	Inferior Epigastric	Direct Anastomosis	Side-to-Side	Ligated
5 years	93.5%	80%	93.3%	100%	95.7%	85.7%
10 years	81.2%	60%	93.3%	100%	95.7%	85.7%

**Table 5:** Adjusted risk analysis of all-cause graft loss Adjusted with donor and recipient age, sex and side of kidney, transplant period, recipient surgeon, operation time, total ischemic time. Abbreviations: aHR, adjusted hazard ratio; CI, confidence interval.

(vs single artery)	All-cause graft loss		
	aHR	95%CI	p-value
End-to-side	3.23	0.43-24.23	0.25
Inferior epigastric artery	0.79	0.091-6.87	0.83
Direct anastomosis	0	0	0.99
Side-to-side	0.13	0.012-1.41	0.093
Ligated	0.44	0.046-4.25	0.47

## Discussion

In Japan, where cadaveric kidney donors are extremely scarce, approximately 90% of kidney transplants are from living-related donors. In addition, the number of transplants between elderly spouses is increasing. Receiving a second transplant is typically challenging; thus, achieving long-term graft survival with a single transplant is crucial. In recent meta-analyses, the long-term graft survival was equivalent between the overall multiple-artery (including all reconstruction techniques) and single-artery groups [1,7]. However, no meta-analyses have analyzed the impact of each specific technique on long-term graft survival. In the present study, the specific reconstruction technique, end-to-side, had inferior long-term graft survival, whereas the other reconstruction methods were comparable to the single-artery group. In some previous studies in the US cohorts, the long-term graft survival of the end-to-side method was markedly worse compared with the other reconstruction methods, which was consistent with our results [3,4]. Furthermore, we found a significantly higher incidence of DGF/SGF in the end-to-side method. It was reported that recipient age and ischemic time could be the predictive factors of DGF [8,9]. However, in the present study, these factors of the end-to-side group did not show much difference. It is assumed that the unique anastomotic configuration of end-to-side inhibits the early graft function recovery and affects long-term graft survival.

It was hypothesized that the insufficient blood supply caused by the configuration of the end-to-side led to short- and long-term perfusion impairment for main and accessory arteries. Two mechanisms for perfusion impairment have been proposed. First, in cases with two renal arteries, the main artery is vulnerable to stenosis because its diameter is relatively small compared with that of a single renal artery, and adding another upstream of the smaller artery to the main artery would create insufficient blood supply to the entire renal cortex, resulting in hypoperfusion of the whole kidney. Second, a smaller artery may not withstand perfusion pressure, leading to stenosis or thrombosis, which could eventually affect the main artery and reduce perfusion in the long term [3]. Paramesh et al. assumed that small accessory arteries in the multi-artery group could be thrombosed after transplantation, which might not have been recognized by routine ultrasound imaging [4].

Notably, no thrombotic complications were observed in any group in the present study, indicating that early surgical complications might not have affected the incidence of DGF/SGF and long-term outcomes. Furthermore, there were no ureteral complications in the multi-arterial subgroup. It was previously believed that urological complications were more common in cases with multiple renal arteries; however, this hypothesis has been refuted in recent studies [10,11]. Notably, the anastomotic site of the reconstructed arteries (internal, external, or common iliac arteries) does not appear to affect long-term survival; however, this was beyond the scope of the present study [12].

Ensuring sufficient blood flow to the entire renal cortex is the fundamental principle of revascularization techniques [13]. Therefore, the discussion of alternative methods to the end-to-side method, aside from direct anastomosis, is warranted. Using the inferior epigastric artery is an option, particularly for the lower pole artery. Its paramount advantage is that it is easily available, has a sufficient length, and can be anastomosed after reperfusion of the main artery and vein [14]. In addition, equivalent short- and long-term outcomes were obtained compared with those of a single artery, including in the present study [3]. We observed that the inferior epigastric artery method was applied to significantly more female donor kidneys than male ones; however, to our knowledge, there is no sexual difference for anatomy of arteries or the applicability of this method [3,15]. Internal iliac artery graft interposition is another option, and the outcomes were equivalent to those of other methods [16]. However, inferior epigastric and internal iliac arteries might not always be available due to calcification. Ligation of a smaller artery that feeds < 5-10% of the renal cortex does not affect long-term graft survival under careful assessment [3,17].

The limitations of this study include its retrospective, single-institution design and small sample size. Immunological and immunosuppressive factors were not analyzed in this study; however, the cumulative incidence of acute rejection and other recipient-related baseline variables were similar between the groups. Therefore, we believe that immunological risks were equally distributed and did not affect our results. However, further multicenter, large-volume studies comparing each specific reconstruction technique with a single artery are warranted. We are planning to conduct multicenter collaborative research in Japan to validate the findings of the present study.

## Conclusions

The end-to-side group demonstrated a higher incidence of delayed graft function and inferior 5- and 10-year graft survival compared to other reconstruction methods. The end-to-side method should be reconsidered when alternative methods are available.
